# Bringing the Training Schools into Line

**Published:** 1919-03-29

**Authors:** 


### THE MATRONS' AND SISTERS' DEPARTMENT.

### BRINGING THE TRAINING SCHOOLS INTO LINE.

The nursing profession has definitely reached a stage in which its most pressing need is to level up all the schools for pupil nurses to a minimum standard of efficiency. This stage has not been reached without years of patient, sometimes it seemed hopeless, labour on the part of far-seeing women. There were times when agreement on the basic principles of a nurse's education seemed unattainable. Ideals differed widely. Various methods of training were on trial. The extraordinary isolation of institution life tended to bar concerted action. The time was not ripe. Then quite suddenly, so it seemed, the training-schools awoke to a perception of the aims and methods they held in common instead of concentrating as they had hitherto done on the local, often purely accidental points of difference between one system and another. They began to stand shoulder to shoulder, to compare and exchange views, to appropriate one from another plans which were hopeful because they had been found to work well. From this stage to the adoption of something like agreement on the main principles of training was but a step, and it has been already taken. The first-class training-schools for nurses at the present day are practically at one in regard to the essential features of a nurse's training. They are turning out every year the women who have set the name of British nurses high among the nations. And by first-class training-schools we would not be held to mean merely the great historic establishments in London and the Provinces and Scotland with ample means for supplying all the educational and structural advantages which are needed by nurses in training. There are first-class training-schools among comparatively small and poor institutions; there are many among Poor-Law institutions; there are some where the very privations are turned to good account in the training by the ability of those in charge.

But below this high level there are many grades, second-class, third-class, no class. Not all the great institutions can be reckoned in the cluster of those pioneer institutions which are abreast of the times. The number of schools which turn out conspicuously second-class nurses is very large. The training is moderately good. It might be, it ought to be, a great deal better. Chances are missed, shortcomings are overlooked. Examinations are conducted on the old perfunctory lines without even outside examiners, and things are very much as they were in the year one. Yet good nurses are turned out and the tone, which is the most vital matter after all, is kept up. These are not first-class, but a few slight alterations would place them in the front rank. They are all ready, some are eager to get there.

The best that can be said of third-class training-schools is that there are fewer of them than formerly, but we have continual evidence of their existence. Badly managed, without proper system, extravagant in results but parsimonious in details, under the dominion of narrow-minded men and women, these institutions own no spur but the dread of failing to get material for training, and are accessible only to threats on the part of their employees. The commercially-minded nurse is manufactured in these establishments. She hears little during her training which can elevate her, and she grows to distrust talk about vocation which in her eyes is made to appear that particular virtue in nurses which makes them easy victims of exploitation.

There are countless institutions which belong to the no-class category. Many of them ought not to be allowed to train nurses at all. Many might do good service if restricted within due limits and helped to assume the office of preparing probationers for serious training. The spurious nurses, the half-trained, the thoroughly inefficient women who are to be encountered in most nursing homes and pose as trained nurses in private practice, spring from the no-class training-centres.

Can anything except legislation bring the laggards into line? Remember it is not a question now of finding out how best to train a nurse. That has been discovered, at any rate as regards the broad lines. It is a question of preventing the education of the nurse from being rendered futile, incomplete, disastrous, by the incompetence of managers. In the public interest ought not a spur to be applied to the laggards?

The College of Nursing has slowly developed all the machinery needed to help the backward training-centres, and in no direction is its help likely to be more fertile for good than in that of subsidising and training sister-tutors for these tasks. But without a definite mandate from the State its activities will be restricted within narrow bounds. The moment has come for getting definite pressure exerted in the training of nurses, and this in the public interest.

